# *α *_2_-Adrenoceptors: Challenges and Opportunities—Enlightenment from the Kidney

**DOI:** 10.1155/2020/2478781

**Published:** 2020-04-29

**Authors:** William A. Pettinger, Edwin K. Jackson

**Affiliations:** ^1^Division of Clinical Pharmacology (Retired), University of Texas Southwestern Medical School (Retired), Bonita Springs, FL, USA; ^2^University of Pittsburgh School of Medicine, Department of Pharmacology and Chemical Biology, 100 Technology Drive, Room 514, Pittsburgh, PA 15219, USA

## Abstract

It was indeed a Don Quixote-like pursuit of the mechanism of essential hypertension when we serendipitously discovered *α*_2_-adrenoceptors (*α*_2_-ARs) in skin-lightening experiments in the frog. Now *α*_2_-ARs lurk on the horizon involving hypertension causality, renal denervation for hypertension, injury from falling in the elderly and prazosin's mechanism of action in anxiety states such as posttraumatic stress disorder (PTSD). Our goal here is to focus on this horizon and bring into clear view the role of *α*_2_-AR-mediated mechanisms in these seemingly unrelated conditions. Our narrative begins with an explanation of how experiments in isolated perfused kidneys led to the discovery of a sodium-retaining process, a fundamental mechanism of hypertension, mediated by *α*_2_-ARs. In this model system and in the setting of furosemide-induced sodium excretion, *α*_2_-AR activation inhibited adenylate cyclase, suppressed cAMP formation, and caused sodium retention. Further investigations led to the realization that renal *α*_2_-AR expression in hypertensive animals is elevated, thus supporting a key role for kidney *α*_2_-ARs in the pathophysiology of essential hypertension. Subsequent studies clarified the molecular pathways by which *α*_2_-ARs activate prohypertensive biochemical systems. While investigating the role of *α*_1_-adrenoceptors (*α*_1_-ARs) versus *α*_2_-ARs in renal sympathetic neurotransmission, we noted an astonishing result: in the kidney *α*_1_-ARs suppress the postjunctional expression of *α*_2_-ARs. Here, we describe how this finding relates to a broader understanding of the role of *α*_2_-ARs in diverse disease states. Because of the capacity for qualitative and quantitative monitoring of *α*_2_-AR-induced regulatory mechanisms in the kidney, we looked to the kidney and found enlightenment.

## 1. Background

While pursuing the hidden antihypertensive mechanism of methyldopa (Aldomet) [1, 2], we examined the effects of methyldopa's metabolites methyldopamine and methylnorepinephrine on reversing melanocyte stimulating hormone- (MSH-) induced darkening of frog (*Rana pipiens*) skin. In these experiments, the skin of the rear thigh of *Rana pipiens* was draped over a 1.2 cm cylinder and light reflectance from a parallel light source was quantified by a photometer; then, MSH was applied. Microscopically, MSH treatment causes a rapid migration of tiny black granules from the dense core granules to completely blacken the skin within a few minutes. Epinephrine, via *α*-adrenoceptors, causes a reverse migration of the granules back into the dense core granules and a tremendous increase in the reflected light in several minutes. We found methyldopamine and methylnorepinephrine to be 30 to 100 times as potent as the naturally occurring dopamine and norepinephrine metabolites of DOPA in reversing MSH-induced darkening of frog skin [3]. That was the basis for discovery of *α*_2_-ARs and the functional classification of *α*-adrenoceptors [4, 5]. Since those early days, three human *α*_2_-AR subtypes, referred to as *α*_2A_-AR, *α*_2B_-AR, and *α*_2C_-AR, have been cloned. Despite having only approximately 50% amino acid sequence identity, these three *α*_2_-AR isoforms have similar structures, pharmacology, and signaling mechanisms, yet differ in tissue distribution (see Aantaa and coworkers [6] for review).

**Figure 1:**
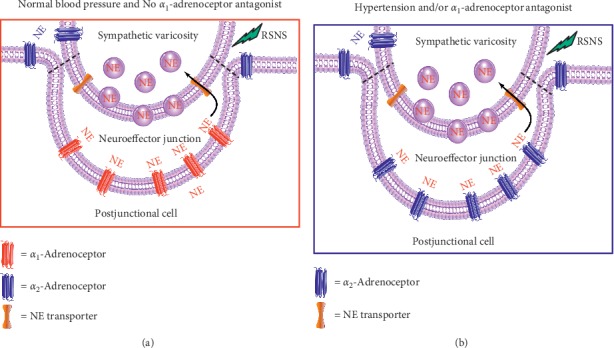
Distribution of *α*-adrenoceptors. Illustration summarizes our conclusion that normally *α*_1_-ARs dominate the postjunctional membrane in the neuroeffector junction (a); however, following chronic treatment with an *α*_1_-AR antagonist or in genetic hypertension, *α*_2_-ARs become the dominant *α*-adrenoceptor subtype residing within the postjunctional membrane (b). In both cases, *α*_2_-ARs are also localized to the prejunctional and extrajunctional membranes. This model was tested using subpressor levels of renal sympathetic nerve stimulation (RSNS) with sodium excretion as the outcome measure and, therefore, applies to sympathetic regulation of sodium reabsorption by renal epithelial cells; however, we hypothesize that similar changes in *α*_2_-ARs may occur in the renal vasculature and may contribute to sodium retention and hypertension. The dotted line (-----) denotes a diffusion barrier that hampers the entry of norepinephrine (NE) into the neuroeffector junction.

Subsequent to the skin-lightening experiments, a large population of *α*_2_-ARs were discovered in normal kidneys [7], the function of which was unknown. An excess of these receptors was found in kidneys of all genetically hypertensive rat strains and further augmented when rats ingested a high-sodium diet [8–10]. Overexpression of *α*_2_-ARs was also present in prazosin (selective *α*_1_-AR antagonist) pretreated normal rats [11]; this surplus of *α*_2_-ARs occupied the anatomic and functional domain unique to *α*_1_-ARs in membrane enclosed postjunctional sites (Figure 1). Thus, these extra* α*_2_-ARs mediate sympathetic nerve-induced sodium retention which is ordinarily unique to *α*_1_-ARs. We suggest that similar multiplication and acquisition of increased or unique function of *α*_2_-ARs occur as mechanisms of various clinical phenomena. While we focus here on excessive expression of *α*_2_-ARs and potential roles they might play in hypertension, therapeutic renal denervation, temporary severe orthostasis, and prazosin's efficacy in stress disorders, we also mention the related issue of bone fractures in race horses.

Essential hypertension in humans is due to excess dietary sodium coupled with the very common genetic predisposition to inappropriate retention of sodium by the kidneys. Sympathetic nerve activation induces sodium retention by the kidney, but its role in hypertension causality was unclear, and for good reason. In vivo studies in humans and in animals were rendered irrelevant because of lowering of systemic arterial pressure by sympathetic blocking agents such as quanethidine and by receptor blocking agents such as phentolamine or prazosin. Decrement in perfusion pressure, as in renal artery stenosis, increases tubular reabsorption of sodium by two mechanisms: one is activation of the renin-angiotensin-aldosterone-sodium retaining mechanism; the second one involves increased tubular reabsorption of sodium independent of aldosterone when renal perfusion pressure is reduced and conversely decreased tubular sodium reabsorption when renal pressure is increased (i.e., renal pressure-natriuresis mechanism). Acute reduction of systemic arterial pressure thus interferes with results of studies of these drugs in vivo, so in vitro perfusion techniques were essential to study these mechanisms. However, technical problems of kidney perfusion precluded use of these techniques as well.

Renal excretory function is dependent on high oxygen tension in the perfusate and oncotic effects of albumin to prevent severe edema in isolated, perfused kidneys. Previous studies failed because bubbling of oxygen denatured and precipitated albumin which clogged arterioles. We overcame these technical problems using the reverse phase of pediatric dialysis coils for oxygenation of albumin/ficol (oncotic activity) perfusates which sustained rat kidney functioning for hours [11–13]. For a schematic diagram of the perfusion apparatus, see the article by Smyth and coworkers [12].

In this isolated perfused rat kidney model, we found that subpressor sympathetic nerve activation promoted sodium retention which was blocked by the *α*_1_-AR antagonist prazosin; however, the *α*_2_-AR antagonist yohimbine had no effect. This is not surprising since *α*_1_-ARs are considered the primary postjunctional *α*-AR subtype mediating noradrenergic neurotransmission via a signaling mechanism that involves coupling to phospholipase C via *G*_q/11_ with subsequent production of inositol trisphosphate, diacylglycerol, and calcium release from the endoplasmic or sarcoplasmic reticulum [14]. However, after three days of prazosin administration, prazosin was no longer effective; yohimbine (*α*_2_-AR antagonist) infusion was required to block sympathetic nerve-induced sodium retention [11]. Of particular interest was that *α*_2_-AR density in the kidney had doubled after three days of prazosin administration. Thus, *α*_1_-AR blockade induces synthesis of new *α*_2_-ARs which then occupy the otherwise exclusive postjunctional domain and function of *α*_1_-ARs (Figure 1). Inasmuch as changes in sodium excretion in response to renal nerve stimulation were studied at frequencies and intensities of renal sympathetic nerve stimulation below those causing vascular resistance increases, the synthesis of new *α*_2_-ARs affecting sodium excretion likely occurred in the postjunctional membranes of renal epithelial cells; however, we hypothesize that this phenomenon is more general and occurs in other tissues as well (e.g., vascular tissues). Notably, norepinephrine (NE) infusion did not cause sodium retention. Thus, a barrier encompasses the sympathetic nerve terminal/*α*_1_-AR linkage which impedes access of circulating NE to the innervated *α*_1_-ARs mediating sodium retention. From these observations, it appears that normally functioning *α*_1_-ARs are suppressive to *α*_2_-AR synthesis. In fact, in genetic hypertensive rats where excess *α*_2_-ARs exist, there is a defect in *α*_1_-AR signaling [15]. It should thus be no surprise that overexpression of* α*_2_-ARs in genetic hypertension (or essential hypertension) mediates excess sodium retention as part of the fundamental cause of hypertension [16]. Currently, the mechanism by which *α*_1_-ARs suppress the expression of *α*_2_-ARs is unknown, and it is imperative to investigate this mechanism in future studies. We speculate that activation of the inositol trisphosphate/calcium/diacylglycerol axis by *α*_1_-ARs in the postjunctional cell leads to either reduced production of *α*_2_-ARs, decreased trafficking of *α*_2_-ARs to the postjunctional membrane, or increased degradation of *α*_2_-ARs.

The complexity of *α*_2_-AR-mediated signaling is illustrated further by the contrasting effects of *α*_2_-AR activation on sodium retention/excretion by kidneys under different hormonal influences. For example, vasopressin infusion causes water and *sodium retention* [17], and when this hormone predominates, *α*_2_-AR activation causes sodium excretion. Alternatively, in the presence of furosemide, adenylate cyclase is activated, sodium excretion is increased, and *α*_2_-AR activation induces sodium retention [12, 18]. We suspect that *α*_2_-AR signaling is context dependent in many other tissues, for example, the brain. Most importantly, isolated kidney perfusion techniques were critical in discovery of increased synthesis and redistribution of *α*_2_-ARs which may explain diverse clinical phenomena as described below.

## 2. Hypertension

High blood pressure is arguably the most important public health problem in the world today. If the kidney has an impaired capacity to excrete sodium (i.e., a shift to the right of the pressure-natriuresis curve) and dietary sodium intake is excessive, an increase in arterial pressure is required to cause natriuresis to restore sodium balance [19]. Thus, hypertension is caused mainly by excess dietary sodium coupled with impaired renal excretion of sodium, often genetically determined in rats and in humans. Millions of people in unacculturated environs do not ingest excess salt and do not have hypertension, strokes, obesity, diabetes, and rarely heart attacks or heart failure [20, 21]. An intact sympathetic nervous system supports high blood pressure [22] and several antihypertensive drugs work through inhibition thereof. Renal sympathetic nerves increase sodium retention by two mechanisms: one is *α*_1_-AR-mediated retention of sodium [17, 23] and the second is via *β*-adrenoceptor activation of the renin-aldosterone-sodium retaining mechanism [24–28].

Thus, it is remarkable that *α*_1_-AR and *β*-adrenoceptor blocking agents, when used alone, have very low efficacy during long-term usage in hypertension treatment. Why? For prazosin, the reason is that excess *α*_2_-ARs and new function thereof occur with blockade of *α*_1_-ARs [11] for three days of treatment with prazosin (Figure 1). In this context, prazosin is remarkably effective for several days in lowering blood pressure [29, 30] and relief of symptoms in congestive heart failure. However, the substitution of *α*_2_-ARs functionally for *α*_1_-ARs impairs the efficacy of prazosin. We suspect that similar excess and translocation of *α*_2_-ARs occur on blood vessels, especially on veins, the reservoir of blood needed to sustain cardiac output, and hence blood pressure, in the upright position.

The low antihypertensive efficacy of *β*-adrenoceptor blockers when used alone is because the dominant controller of renin release is a pressor-volume mechanism [31] independent of *β*-adrenoceptors. However, when the sympathetic nervous system is reflexly activated by peripheral acting antihypertensive drugs, *β*-adrenoceptor blockade of excess renin release is a powerful blood pressure lowering mechanism [26–28]. Extensive blood pressure lowering in severely hypertensive patients leads to passive tubular retention of sodium that can be overpowering leading to severe edema, even cardiac tamponade [32], details of which are described by Pettinger [33]. Moreover, severe preglomerular arteriolar hypertrophy coupled with the magnitude of blood pressure reduction decreases glomerular filtration rate causing uremia. This syndrome is known as the pseudorenal artery stenosis syndrome (PRASS) [33]. It is important because extremely elevated serum creatinine and urea in this syndrome could precipitate needless hemodialysis for the remainder of a patient's life. By simply adjusting medications, kidney function can be restored. It is of particular note that reversal of arteriolar hypertrophy should be a goal of hypertension treatment [33].

With regard to hypertension, sex is an important biological variable with premenopausal women having a lower blood pressure compared to age-matched men [34]. Interestingly, we found that one gene for the overexpression of *α*_2_-AR-specific mRNA is on the Y-chromosome and one or more are on somatic chromosome(s) [35, 36]. This relationship is one additional association supportive of overexpression of *α*_2_-ARs in causality of human hypertension. Incidentally, in order for increased *α*_2_-ARs to sustain a tonal retention of sodium, our model presumes an endogenous factor stimulatory to a renal adenylate cyclase pool/isoform that is linked to sodium excretion by the kidneys. Who will discover this remarkable substance?

**Figure 2:**
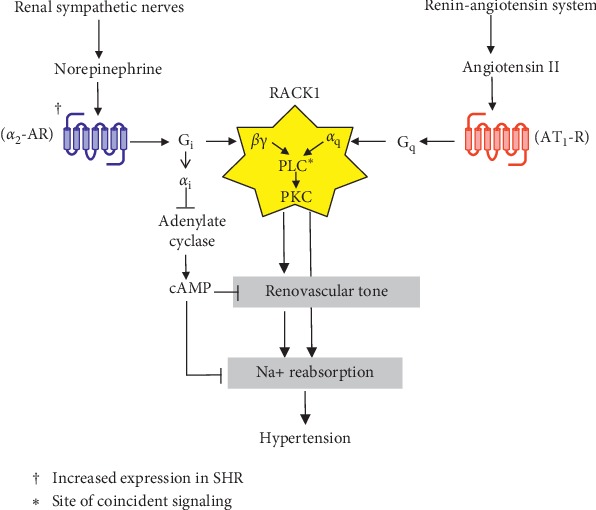
Signaling mechanisms in the SHR renal microcirculation. Renal sympathetic nerves release norepinephrine which stimulates *α*_2_-adrenoceptors (*α*_2_-ARs) in renal vascular smooth muscle cells, thus leading to dissociation of *G*_*i*_ and release of *α*_*i*_ and *βγ* subunits. Angiotensin II engages type 1 angiotensin II receptors (AT_1_-Rs) which results in the release of *α*_q_ from *G*_q_. *βγ* subunits arising from G_*i*_-coupled *α*_2_-ARs bind receptor for activated C kinase 1 (RACK1) and localize this scaffolding protein to the cell membrane. At the cell membrane, RACK1 also binds phospholipase C (PLC) and protein kinase C (PKC), and PLC binds *α*_q_. Together, these interactions result in an efficient signaling complex in which activation of PLC by *α*_q_ is enhanced by the simultaneous binding of *βγ* subunits to PLC. Thus, PLC serves as a coincident detector, whereas RACK1 functions here to bring together the stimulating components of this coincident signaling mechanism. This coincident signaling mechanism is further amplified by the fact that RACK1 localizes PLC with PKC, thus facilitating the activation of PKC, which mediates contraction of vascular smooth muscle cells. In addition to *βγ*-mediated signaling, release of *α*_*i*_ by *α*_2_-ARs inhibits the adenylate cyclase/cAMP pathway, which further increases contraction of vascular smooth muscle cells. Because of the increased pool of G_*i*_-coupled *α*_2_-ARs, both the *α*_*i*_-mediated and *βγ*-mediated mechanisms are more engaged in the SHR renal microvasculature, thus leading to renal vasoconstriction, sodium retention, and hypertension. The model was tested using pressor levels of angiotensin II with renovascular responses (or in some experiments contractile responses to isolated preglomerular vascular smooth muscle cells) as the outcome measure and, therefore, applies to sympathetic regulation of renal vascular smooth muscle cells; however, we hypothesize that similar coincident signaling involving *α*_2_-ARs may occur in renal epithelial cells and may contribute directly to sodium retention and hypertension independent of renovascular changes.

Further evidence for an important role of renal *α*_2_-ARs in hypertension is provided by studies in the spontaneously hypertensive rat (SHR), a genetic model of hypertension with excessive expression of renal *α*_2_-ARs. Despite normal plasma and kidney levels of angiotensin II, as well as normal expression of angiotensin II type 1 receptors (AT_1_Rs) in the kidney, this model of genetic hypertension is fully dependent on the renin-angiotensin system for development and maintenance of an elevated blood pressure [37]. Why? The reason is in part due to the increased sensitivity of the renal microcirculation to angiotensin II-induced vasoconstriction [37–42]. Importantly, *α*_2_-ARs are G_*i*_-coupled receptors that upon activation release large quantities of *βγ* G-protein subunits from the G_*i*_ ternary complex (i.e., *α*_*i*_*βγ*), and AT_1_Rs are Gq-coupled receptors that upon stimulation release *α*_q_ G-protein subunits from the G_q_ ternary complex (i.e., *α*_q_*βγ*). As discussed by Selbie and Hill [43] and Philip and coworkers [44], when *βγ* subunits bind to specific isoforms of phospholipase C (PLC), the ability of *α*_q_ to activate the enzymatic activity of PLC is remarkably enhanced. PLC, in part via activation of protein kinase C (PKC), then promotes renal vasoconstriction. In SHR kidneys, but not kidneys from genetically normotensive rats, this signaling “cross-talk” or “coincident signaling” is engaged when *α*_2_-ARs are stimulated with the selective agonist UK 14,304 and AT_1_Rs are simultaneously activated with angiotensin II [45–48]. Likely, the enhanced expression of renal *α*_2_-ARs in SHR kidneys provides an enlarged membrane-localized pool of *βγ* subunits which tethers the scaffolding protein receptor for activated C kinase 1 (RACK1) to the cell membrane [49]. RACK1 then organizes an efficient signaling complex (Figure 2) consisting of *βγ* subunits released from *α*_2_-ARs, *α*_q_ subunits released from AT_1_Rs, PLC, and PKC [49]. The importance of *α*_2_-AR/G_*i*_-mediated release of *βγ* in this mechanism is highlighted by recent findings that G_*i*_-coupled receptors are particularly effective in releasing active *βγ* subunits. In contrast, other G-proteins (e.g., *G*_q_ and *G*_13_) tend to restrain the signaling capabilities of *βγ* subunits released from their corresponding *αβγ* complexes [50]. In addition to the coincidence signaling mechanism described above, *α*_2_-AR-mediated release of *α*_*i*_ from the *α*_*i*_*βγ* ternary complex may also contribute to the enhanced renovascular response to angiotensin II in SHR kidneys (Figure 2). Indeed, *α*_*i*_ inhibits the adenylate cyclase/cAMP pathway [51], which may explain why the ability of prostacyclin (activates adenylate cyclase) to inhibit angiotensin II-induced renal vasoconstriction is impaired in SHR kidneys [52, 53]. Taken together, the evidence suggests that *α*_2_-ARs importantly contribute to the enhanced vasoconstrictive effects of angiotensin II in SHR kidneys. Further support for this model is that inhibition of *α*_2_-AR signaling with pertussis toxin, a toxin that blocks G_*i*_-mediated signaling [54], normalizes renovascular responses to angiotensin II in SHR kidneys and chronically reverses hypertension in SHR [55, 56], despite the fact that pertussis toxin activates renin release [57]. The model shown in Figure 2 was tested using pressor levels of angiotensin II with renovascular responses (or in some experiments contractile responses to isolated preglomerular vascular smooth muscle cells) as the outcome measure and, therefore, applies to sympathetic regulation of renal vascular smooth muscle cells; however, we hypothesize that similar coincident signaling involving *α*_2_-ARs may occur in renal epithelial cells and may contribute directly to sodium retention and hypertension independent of renovascular changes (Figure 2).

## 3. Renal Denervation for Hypertension

To the extent that *α*_1_-AR-mediated sodium retention is a basic mechanism of hypertension, destruction of sympathetic nerves to the kidney should lower blood pressure. Indeed, catheter-based renal denervation (RDN) has shown promise as a treatment for resistant hypertension [58, 59]. However, the phase 3 Symplicity HTN-3 trial in patients with resistant hypertension failed to achieve its primary efficacy endpoint; this outcome has dampened initial enthusiasm regarding the utility of RDN [60]. Why the disappointing results? Several hypotheses have been proposed including operator inexperience, suboptimal design of the ablation catheter, and need for better patient selection [61]. However, another consideration is that perhaps like prazosin, RDN prevents the tonic influence of *α*_1_-ARs to suppress the synthesis of *α*_2_-ARs. Thus, RDN likely would upregulate the expression of renal *α*_2_-ARs in the renal sympathetic neuroeffector junction. Upregulated *α*_2_-ARs may express agonist-independent (constitutive or intrinsic) activity [62] or could be activated by circulating catecholamines. In support of this hypothesis, studies by Vallon and coworkers [63] revealed that in the setting of subacute RDN, activation of *α*_2_-ARs sensitizes kidneys to the detrimental effects of angiotensin II and nitric oxide synthase inhibition.

## 4. Injury from Falls in the Elderly and Orthostatic Hypotension

Falling is a frequent cause of serious injury and death in the elderly and orthostatic hypotension often causes falling. With prazosin, blood pressure drops in the standing position because of blockade of postsynaptic *α*_1_-ARs. In this context, postural hypotension can be severe during the first few days of prazosin treatment or reinitiation of treatment [29, 30]. Prazosin and other *α*_1_-AR antagonists are used frequently to reduce urethral tone in prostatic obstruction in elderly men, in stress disorders and in treatment of hypertension. Fortunately, severe orthostasis is temporary, and we attribute reversal thereof to stimulation of newly expressed *α*_2_-ARs that occupy the otherwise exclusive postjunctional domain of *α*_1_-ARs. The exaggerated temporary orthostasis is due to two mechanisms: one is reduced retention of sodium and water by the kidney causing volume contraction in venous reservoirs; the second mechanism is due to a decrease in venous tone which occurs during the time interval before multiplication and assumption of *α*_1_-AR function by newly synthesized *α*_2_-ARs.

Orthostatic hypotension is often severe in patients with excess circulating NE in pheochromocytoma, an apparent oxymoron. In the past this was attributed to volume contraction from venoconstriction which could, of course, be a contributing factor. However, this hypotension can also be explained with the model of the sympathetic nerve/*α*-adrenoceptor junction and its protective barrier discovered in the isolated kidney perfusion model. In this regard, pioneering work by Solomon Langer [64] and Klaus Starke [65] demonstrated the existence of prejunctional *α*_2_-ARs that are inhibitory to NE release. In pheochromocytoma, high levels of circulating NE have access to these presynaptic *α*_2_-ARs that are inhibitory to NE release. Thus, *α*_2_-AR-mediated inhibition of NE release in the standing position may also contribute to orthostatic hypotension in pheochromocytoma.

## 5. Posttraumatic Stress Disorder (PTSD) and Anxiety

Propranolol [66–69], prazosin [70–72], and combinations thereof [68] are widely used drugs for treatment of PTSD. Even so, management of PTSD remains problematic, and novel pharmacotherapies are badly needed. One approach to develop new PTSD drugs would be to elucidate the mechanisms of action by which currently available drugs relieve symptoms and exploit that knowledge to advance better treatments. In this regard, even though prazosin is often used to manage PTSD, its mechanism of action remains obscure. Interestingly, in the brain *α*_2_-ARs mediate suppression of anxiety such that withdrawal of treatment with *α*_2_-AR agonists (e.g., clonidine and methyldopa) precipitates anxiety and sleeplessness [73, 74]. Since prazosin administration increases *α*_2_-ARs in the kidney which assumes additional activity in sodium retention, a similar phenomenon may occur in the brain. Thus, we hypothesize that the efficacy of prazosin in PTSD is mediated at least partially by upregulation of *α*_2_-ARs. This is a testable hypothesis that if proven true would indicate a new avenue of research for effective PTSD treatments.

## 6. Furosemide in Race Horse Deaths: *α*_2_-AR Blockers to the Rescue

In the same laboratory where *α*_2_-ARs were discovered along with their substitution for *α*_1_-ARs following prazosin administration, microdissection/microchemical techniques simultaneously pursued correlates of the physiological studies involving the adenylate cyclase inhibitory role of excess *α*_2_-ARs. Thus, the renal adenylate cyclase stimulatory mechanism of furosemide was discovered in which furosemide was shown to increase urinary cyclic AMP excretion by approximately 5-fold [12]. Although distinct from furosemide's generally accepted mechanism of diuretic activity, i.e., inhibition of the Na^+^-K^+^-2Cl^−^ symporter (see Jackson [75] for review), it is interesting that activation of G_*i*_-linked *α*_2_-ARs reduces both cyclic AMP and sodium excretion in furosemide-treated kidneys.

Normally, the operation of the Na^+^-K^+^-2Cl^−^ symporter in the thick ascending limb provides for a transepithelial potential difference (PD) of about 10 mV (lumen positive with respect to the interstitium). This lumen positive PD drives the reabsorption of Ca^2+^ (which being a cation is repulsed by a positive PD) via the paracellular pathway. Loop diuretics, such as furosemide, bind to and inhibit the Na^+^-K^+^-2Cl^−^ symporter, which destroys the lumen positive PD and thereby reduces the driving force for Ca^2+^ reabsorption. This is why loop diuretics increase Ca^2+^ excretion leading to hypocalcemia [75]. Low serum Ca^2+^ levels stimulate parathyroid hormone (PTH) release, which mobilizes Ca^2+^ from bone leading to decreased bone density. However, the aforementioned mechanism does not rule out the possibility that furosemide increases Ca^2+^ mobilization from bone via an additional, and more direct, mechanism involving stimulation of adenylate cyclase in osteoblasts/osteoclasts, similar to PTH. Currently, huge doses of furosemide are used repeatedly in race horses to prevent lung hemorrhage. Via the aforementioned mechanisms, furosemide in race horses would be expected to absorb excess calcium from bone causing osteoporosis and predisposition to leg fracture, which may explain the large number of deaths in race horses due to leg fracture. Indeed, loop diuretics are also known to promote osteoporosis in humans [76, 77].

How does loop diuretic-induced bone disease relate to *α*_2_-ARs? Inasmuch as *α*_2_-ARs inhibit the adenylate cyclase/cAMP pathway, one would predict that *α*_2_-AR agonists might reduce osteopenia/osteoporosis. However, as with the kidney, the effects of *α*_2_-AR signaling in bone also appear to be context dependent since the published data suggest that *α*_2_-AR antagonists, rather than agonists, protect against bone loss. For example, female mice with double knockout of *α*_2A_-ARs and *α*_2C_-ARs present a surprising phenotype characterized by (1) increased bone mass; (2) reduced bone resorption; (3) augmented bone formation; (4) increased and better connected and more plate-shaped trabeculae in the femur and vertebrae; (5) increased cortical thickness in the vertebrae; and (6) increased tibial and femoral strength [78]. Moreover, the *α*_2_-AR agonist clonidine stimulates resorptive activity of osteoclasts in culture [78]. Recent studies in humans show that single nucleotide polymorphisms located in the *α*_2A_-AR gene are associated with osteoporosis and significantly increase *α*_2A_-AR mRNA levels in human bone samples by stabilizing mRNA [79]. Together, these findings encourage the pursuit of *α*_2_-AR antagonists for prevention and treatment of osteopenia/osteoporosis, including disease promoted by loop diuretics.

## 7. Conclusion

Because of directly measurable effects of neuroregulatory control mechanisms in the kidney, we discovered a tonal mechanism of *α*_1_-AR suppression of synthesis, redistribution, and function of *α*_2_-ARs that appears applicable to several clinical phenomena. These include the basic mechanism of hypertension, unexpectedly low efficacy of renal denervation for hypertension, severe injury from falling, especially in the elderly, and a mechanism of action for prazosin in stress disorders (PTSD). These kidney experiments also revealed the context-dependent effects of *α*_2_-ARs [80, 81], which are likely due to the complex signaling mechanisms engaged by these receptors. From a public health viewpoint, the causal role of altered *α*_2_-AR regulation in hypertension via retention of excess sodium appears very promising. Millions of people ingesting 20 times the real minimum daily requirement for sodium [33] have no hypertension which is obviously genetically determined; they are salt-resistant. A drug which would convert salt-sensitive hypertension to salt-resistance by interfering selectively with the *α*_2_-AR-sodium retaining mechanism could be paradigm shifting. It could prevent heart attacks and failure, strokes and aortic ruptures, many cases of renal failure and dementias, remarkable goals indeed. Coincidentally, the discovery of *α*_2_-ARs may lead to a possible treatment for osteopenia/osteoporosis, including disease promoted by loop diuretics, thus reducing the incidence of bone fractures in humans and animals. Although not discussed here, potential uses for *α*_2_-AR drugs keep expanding and include glaucoma, ocular hypertension, rosacea, preeclampsia, chronic pain, anesthesia, autonomic dysfunction, attention-deficit hyperactivity disorder, opioid withdrawal, diabetes, neuropsychiatric disorders, and migraines. We conclude that hypotheses concerning potential biologic mechanisms of *α*_2_-ARs are guideposts of major future and continuing discoveries. The discovery of *α*_2_-ARs, their altered regulation, and their potential roles reviewed herein are examples of such guideposts.

## Additional Points

A very recent article by Nemet and workers (Cell. 2020; 180(5):862-877) establishes that phenylacetic acid, a gut microbial metabolite, is absorbed and converted by the liver to phenylacetylglutamine. The authors also show that phenylacetylglutamine is an *α*_2_-adrenoceptor agonist that activates platelet *α*_2_-adrenoceptors to enhance thrombosis potential. In patients, levels of this unique *α*_2_-adrenoceptor agonist associate with heart attacks, strokes, and death. These recent findings reveal yet another critically important role of the *α*_2_-adrenoceptor system in human disease.

## References

[1] Pettinger W., Horwitz D., Spector S., Sjoerdsma A. (1963). Enhancement by methyldopa of tyramine sensitivity in man. *Nature*.

[2] Pettinger W. A., Spector S., Horwitz D., Sjoerdsma A. (1965). Restoration of tyramine pressor responses in reserpine-treated animals by methyldopa and its amine metabolites. *Experimental Biology and Medicine*.

[3] Pettinger W. A. (1977). Unusual alpha adrenergic receptor potency of methyldopa metabolites on melanocyte function. *The Journal of Pharmacology and Experimental Therapeutics*.

[4] Berthelsen S., Pettinger W. A. (1977). A functional basis for classification of *α*-adrenergic receptors. *Life Sciences*.

[5] Pettinger W. A. (1988). This week’s citation classic. *Current Contents*.

[6] Aantaa R., Marjamäki A., Scheinin M. (1995). Molecular pharmacology of *α*_2_-adrenoceptor subtypes. *Annals of Medicine*.

[7] Schmitz J. M., Graham R. M., Sagalowsky A., Pettinger W. A. (1981). Renal *alpha*-1 and *alpha*-2 adrenergic receptors: biochemical and pharmacological correlations. *The Journal of Pharmacology and Experimental Therapeutics*.

[8] Graham R. M., Pettinger W. A., Sagalowsky A., Brabson J., Gandler T. (1982). Renal alpha-adrenergic receptor abnormality in the spontaneously hypertensive rat. *Hypertension*.

[9] Pettinger W. A., Gandler T., Sanchez A., Saavedra J. M. (1982). Dietary sodium and renal *α*_2_-adrenergic receptors in dahl hypertensive rats. *Clinical and Experimental Hypertension. Part A: Theory and Practice*.

[10] Sanchez A., Pettinger W. A. (1981). Dietary sodium regulation of blood pressure and renal *α*_1_- and *α*_2_- receptors in WKY and SH rats. *Life Sciences*.

[11] Smyth D. D., Umemura S., Pettinger W. A. (1986). Renal *α*_2_-adrenergic receptors multiply and mediate sodium retention after prazosin treatment. *Hypertension*.

[12] Smyth D. D., Umemura S., Pettinger W. A. (1984). *α *
_2_-adrenoceptors and sodium reabsorption in the isolated perfused rat kidney. *American Journal of Physiology-Renal Physiology*.

[13] Smyth D. D., Umemura S., Pettinger W. A. (1984). *Alpha*-1 adrenoceptor selectivity of phenoxybenzamine in the rat kidney. *The Journal of Pharmacology and Experimental Therapeutics*.

[14] Docherty J. R. (2019). The pharmacology of *α*1-adrenoceptor subtypes. *European Journal of Pharmacology*.

[15] Jeffries W. B., Yang E., Pettinger W. A. (1988). Renal alpha 1-adrenergic receptor response coupling in spontaneously hypertensive rats. *Hypertension*.

[16] Pettinger W. A. (1987). Renal alpha 2-adrenergic receptors and hypertension. *Hypertension*.

[17] Smyth D. D., Umemura S., Pettinger W. A. (1985). Renal nerve stimulation causes alpha 1-adrenoceptor-mediated sodium retention but not alpha 2-adrenoceptor antagonism of vasopressin. *Circulation Research*.

[18] Pettinger W. A., Umemura S., Smyth D. D., Jeffries W. B. (1987). Renal alpha 2-adrenoceptors and the adenylate cyclase-cAMP system: biochemical and physiological interactions. *American Journal of Physiology-Renal Physiology*.

[19] Guyton A. (1991). Blood pressure control--special role of the kidneys and body fluids. *Science*.

[20] Carvalho J. J., Baruzzi R. G., Howard P. F. (1989). Blood pressure in four remote populations in the INTERSALT Study. *Hypertension*.

[21] Group I. C. R. (1988). Intersalt: an international study of electrolyte excretion and blood pressure. Results for 24 hour urinary sodium and potassium excretion. *BMJ*.

[22] Smithwick R. H., Thompson J. E. (1953). Splanchnicectomy for essential hypertension. *Journal of the American Medical Association*.

[23] DiBona G. F. (1992). Sympathetic neural control of the kidney in hypertension. *Hypertension*.

[24] Campbell W. B., Pettinger W. A., Keeton K., Brooks S. N. (1975). Vasodilating antihypertensive drug-induced aldosterone release--a study of endogenous angiotensin-mediated aldosterone release in the rat. *The Journal of Pharmacology and Experimental Therapeutics*.

[25] Keeton T. K., Pettinger W. A. (1979). The dominance of adrenergic mechanisms in mediating hypotensive drug-induced renin release in the conscious rat. *The Journal of Pharmacology and Experimental Therapeutics*.

[26] Pettinger W. A., Campbell W. B., Keeton K. (1973). Adrenergic component of renin release induced by vasodilating antihypertensive drugs in the rat. *Circulation Research*.

[27] Pettinger W. A., Keeton K. (1975). Altered renin release and propranolol potentiation of vasodilatory drug hypotension. *Journal of Clinical Investigation*.

[28] Pettinger W. A., Mitchell H. C. (1975). Renin release, saralasin and the vasodilator-beta-blocker drug interaction in man. *New England Journal of Medicine*.

[29] Graham R. M., Thornell I. R., Gain J. M., Bagnoli C., Oates H. F., Stokes G. S. (1976). Prazosin: the first-dose phenomenon. *Bmj*.

[30] Koch-Weser J., Graham R. M., Pettinger W. A. (1979). Prazosin. *New England Journal of Medicine*.

[31] Keeton T. K., Campbell W. B. (1980). The pharmacologic alteration of renin release. *Pharmacological Reviews*.

[32] Pettinger W. A. (1980). Minoxidil and the treatment of severe hypertension. *The New England Journal of Medicine*.

[33] Pettinger W. A. (2017). Hypertensionʼs 3 dilemmas and 3 solutions. *Journal of Cardiovascular Pharmacology*.

[34] Dubey R., Oparil S., Imthurn B., Jackson E. K. (2002). Sex hormones and hypertension. *Cardiovascular Research*.

[35] Gong G., Dobin A., McArdle S., Sun L., Johnson M. L., Pettinger W. A. (1994). Sex influence on renal alpha 2-adrenergic receptor density in the spontaneously hypertensive rat. *Hypertension*.

[36] Gong G., Pettinger W. A. (1994). Does the kidney play a role in the sexual dimorphism of blood pressure in SHR?. *Hypertension*.

[37] Li P., Jackson E. K. (1989). Enhanced slow-pressor response to angiotensin II in spontaneously hypertensive rats. *The Journal of Pharmacology and Experimental Therapeutics*.

[38] Kost C. K., Jackson E. K. (1993). Enhanced renal angiotensin II subtype 1 receptor responses in the spontaneously hypertensive rat. *Hypertension*.

[39] Kost C. K., Herzer W. A., Li P., Jackson E. K. (1994). Vascular reactivity to angiotensin II is selectively enhanced in the kidneys of spontaneously hypertensive rats. *The Journal of Pharmacology and Experimental Therapeutics*.

[40] Vyas S. J., Jackson E. K. (1995). Angiotensin II: enhanced renal responsiveness in young genetically hypertensive rats. *The Journal of Pharmacology and Experimental Therapeutics*.

[41] Kost C. K., Herzer W. A., Li P. (1996). Angiotensin II-induced structural and functional alterations in spontaneously hypertensive rat kidney. *American Journal of Physiology-Renal Physiology*.

[42] Kost C. K., Li P., Williams D. S., Jackson E. K. (1998). Renal vascular responses to angiotensin II in conscious spontaneously hypertensive and normotensive rats. *Journal of Cardiovascular Pharmacology*.

[43] Selbie L. A., Hill S. J. (1998). G protein-coupled-receptor cross-talk: the fine-tuning of multiple receptor-signalling pathways. *Trends in Pharmacological Sciences*.

[44] Philip F., Kadamur G., Silos R. G., Woodson J., Ross E. M. (2010). Synergistic activation of phospholipase C-*β*3 by G*α*_q_ and G*βγ* describes a simple two-state coincidence detector. *Current Biology*.

[45] Jackson E. K., Herzer W. A., Kost C. K., Vyas S. J. (2001). Enhanced interaction between renovascular *α*2-adrenoceptors and angiotensin II receptors in genetic hypertension. *Hypertension*.

[46] Jackson E. K., Gillespie D. G., Zhu C., Ren J., Zacharia L. C., Mi Z. (2008). *α*
_2_-Adrenoceptors enhance angiotensin II-induced renal vasoconstriction. *Hypertension*.

[47] Jackson E. K., Gao L., Zhu C. (2005). Mechanism of the vascular angiotensin II/*α*2-adrenoceptor interaction. *Journal of Pharmacology and Experimental Therapeutics*.

[48] Gao L., Zhu C., Jackson E. K. (2003). *α*
_2_-Adrenoceptors potentiate angiotensin II- and vasopressin-induced renal vasoconstriction in spontaneously hypertensive rats. *Journal of Pharmacology and Experimental Therapeutics*.

[49] Zhu X., Jackson E. K. (2017). RACK1 regulates angiotensin II-induced contractions of SHR preglomerular vascular smooth muscle cells. *American Journal of Physiology-Renal Physiology*.

[50] Cervantes-Villagrana R. D., Adame-García S. R., García-Jiménez I. (2019). G*βγ* signaling to the chemotactic effector P-REX1 and mammalian cell migration is directly regulated by G*α*q and G*α*13 proteins. *Journal of Biological Chemistry*.

[51] Burns T. W., Langley P. E., Terry B. E. (1981). Pharmacological characterizations of adrenergic receptors in human adipocytes. *Journal of Clinical Investigation*.

[52] Jackson E. K., Herzer W. A. (1993). Angiotensin II/prostaglandin I2 interactions in spontaneously hypertensive rats. *Hypertension*.

[53] Jackson E. K., Herzer W. A. (1994). Defective modulation of angiotensin II-induced renal vasoconstriction in hypertensive rats. *Hypertension*.

[54] Katada T. (2012). The inhibitory G protein Gi identified as pertussis toxin-catalyzed ADP-ribosylation. *Biological and Pharmaceutical Bulletin*.

[55] Jackson E. K., Herzer W. A., Vyas S. J., Kost C. K. (1999). Angiotensin II-induced renal vasoconstriction in genetic hypertension. *The Journal of Pharmacology and Experimental Therapeutics*.

[56] Kost C. K., Herzer W. A., Li P. J., Jackson E. K. (1999). Pertussis toxin-sensitive G-proteins and regulation of blood pressure in the spontaneously hypertensive rat. *Clinical and Experimental Pharmacology and Physiology*.

[57] Pedraza-Chaverrí J., Alatorre-González M. C., Ibarra-Rubio M. E., Carlos Peña J., García-Sáinz J. A. (1984). Effect of pertussis toxin on the adrenergic regulation of plasma renin activity. *Life Sciences*.

[58] Kiuchi M. G., Esler M. D., Fink G. D. (2019). Renal denervation update from the International Sympathetic Nervous System Summit. *Journal of the American College of Cardiology*.

[59] Townsend R. R., Sobotka P. A. (2018). Catheter-based renal denervation for hypertension. *Current Hypertension Reports*.

[60] Al Raisi S. I., Pouliopoulos J., Swinnen J., Thiagalingam A., Kovoor P. (2019). Renal artery denervation in resistant hypertension: the good, the bad and the future. *Heart, Lung and Circulation*.

[61] Dasgupta I., Sharp A. S. P. (2019). Renal sympathetic denervation for treatment of hypertension. *Current Opinion in Nephrology and Hypertension*.

[62] Pauwels P., Tardif S. (2002). Enhanced stability of wild-type and constitutively active *α* 2A -adrenoceptors by ligands with agonist, silent and inverse agonist properties. *Naunyn-Schmiedeberg’s Archives of Pharmacology*.

[63] Vallon V., Peterson O. W., Gabbai F. B., Blantz R. C., Thomson S. C. (1995). Interactive control of renal function by *α*2-adrenergic system and nitric oxide. *Journal of Cardiovascular Pharmacology*.

[64] Langer S. Z. (1977). Sixth Gaddum Memorial Lecture National Institute for Medical Research, Mill Hill, January 1977 presynaptic receptors and their role in the regulation of transmitter release. *British Journal of Pharmacology*.

[65] Starke K. (1977). Regulation of noradrenaline release by presynaptic receptor systems. *Reviews of Physiology, Biochemistry and Pharmacology*.

[66] Strawn J. R., Geracioti T. D. (2008). Noradrenergic dysfunction and the psychopharmacology of posttraumatic stress disorder. *Depression and Anxiety*.

[67] Bastien D. L. (2010). Pharmacological treatment of combat-induced PTSD: a literature review. *British Journal of Nursing (Mark Allen Publishing)*.

[68] Shad M. U., Suris A. M., North C. S. (2011). Novel combination strategy to optimize treatment for PTSD. *Human Psychopharmacology: Clinical and Experimental*.

[69] Giustino T. F., Fitzgerald P. J., Maren S. (2016). Revisiting propranolol and PTSD: memory erasure or extinction enhancement?. *Neurobiology of Learning and Memory*.

[70] Breen A., Blankley K., Fine J. (2017). The efficacy of prazosin for the treatment of posttraumatic stress disorder nightmares in U.S. military veterans. *Journal of the American Association of Nurse Practitioners*.

[71] Berardis D., Marini S., Serroni N. (2015). Targeting the noradrenergic system in posttraumatic stress disorder: a systematic review and meta-analysis of prazosin trials. *Current Drug Targets*.

[72] Simon P. Y. R., Rousseau P.-F. (2017). Treatment of post-traumatic stress disorders with the alpha-1 adrenergic antagonist prazosin. *The Canadian Journal of Psychiatry*.

[73] Reid J. L., Campbell B. C., Hamilton C. A. (1984). Withdrawal reactions following cessation of central alpha-adrenergic receptor agonists. *Hypertension*.

[74] Horwitz D., Pettinger W. A., Orvis H., Thomas R. E., Sjoerdsma A. (1967). Effects of methyldopa in fifty hypertensive patients. *Clinical Pharmacology & Therapeutics*.

[75] Jackson E. K., Brunton L. L. (2018). Drugs affecting renal excretory function. *Goodman & Gilman’s the Pharmacological Basis of Therapeutics*.

[76] Lim L. S., Fink H. A., Kuskowski M. A. (2008). Loop diuretic use and increased rates of hip bone loss in older men. *Archives of Internal Medicine*.

[77] Paik J. M., Rosen H. N., Gordon C. M., Curhan G. C. (2016). Diuretic use and risk of vertebral fracture in women. *The American Journal of Medicine*.

[78] Fonseca T. L., Jorgetti V., Costa C. C. (2011). Double disruption of *α*_2A_- and *α*_2C_-adrenoceptors results in sympathetic hyperactivity and high-bone-mass phenotype. *Journal of Bone and Mineral Research*.

[79] Mlakar V., Jurkovic Mlakar S., Zupan J., Komadina R., Prezelj J., Marc J. (2015). ADRA2A is involved in neuro-endocrine regulation of bone resorption. *Journal of Cellular and Molecular Medicine*.

[80] Umemura S., Smyth D. D., Pettinger W. A. (1986). Regulation of renal cellular cAMP levels by prostaglandins and alpha 2-adrenoceptors: microdissection studies. *Kidney International*.

[81] Umemura S., Smyth D. D., Pettinger W. A. (1986). Alpha 2-adrenoceptor stimulation and cellular cAMP levels in microdissected rat glomeruli. *Am J Physiol*.

